# Traditional Chinese medicine improved diabetic kidney disease through targeting gut microbiota

**DOI:** 10.1080/13880209.2024.2351946

**Published:** 2024-05-17

**Authors:** Xia-Qing Wu, Lei Zhao, Yan-Long Zhao, Xin-Yao He, Liang Zou, Ying-Yong Zhao, Xia Li

**Affiliations:** a Faculty of Life Science & Medicine, Northwest University, Xi’an, Shaanxi, China; b Department of General Practice, Xi’an International Medical Center Hospital, Xi’an, Shaanxi, China; c School of Food and Bioengineering, Chengdu University, Chengdu, Sichuan, China; d School of Pharmacy, Zhejiang Chinese Medical University, Hangzhou, Zhejiang, China

**Keywords:** Chronic kidney disease, intestinal epithelial cell barrier

## Abstract

**Context:**

Diabetic kidney disease (DKD) affects nearly 40% of diabetic patients, often leading to end-stage renal disease that requires renal replacement therapies, such as dialysis and transplantation. The gut microbiota, an integral aspect of human evolution, plays a crucial role in this condition. Traditional Chinese medicine (TCM) has shown promising outcomes in ameliorating DKD by addressing the gut microbiota.

**Objective:**

This review elucidates the modifications in gut microbiota observed in DKD and explores the impact of TCM interventions on correcting microbial dysregulation.

**Methods:**

We searched relevant articles from databases including Web of Science, PubMed, ScienceDirect, Wiley, and Springer Nature. The following keywords were used: diabetic kidney disease, diabetic nephropathy, gut microbiota, natural product, TCM, Chinese herbal medicine, and Chinese medicinal herbs. Rigorous criteria were applied to identify high-quality studies on TCM interventions against DKD.

**Results:**

Dysregulation of the gut microbiota, including *Lactobacillus*, *Streptococcus*, and *Clostridium*, has been observed in individuals with DKD. Key indicators of microbial dysregulation include increased uremic solutes and decreased short-chain fatty acids. Various TCM therapies, such as formulas, tablets, granules, capsules, and decoctions, exhibit unique advantages in regulating the disordered microbiota to treat DKD.

**Conclusion:**

This review highlights the importance of targeting the gut-kidney axis to regulate microbial disorders, their metabolites, and associated signaling pathways in DKD. The Qing-Re-Xiao-Zheng formula, the Shenyan Kangfu tablet, the Huangkui capsule, and the Bekhogainsam decoction are potential candidates to address the gut-kidney axis. TCM interventions offer a significant therapeutic approach by targeting microbial dysregulation in patients with DKD.

## Introduction

Diabetic kidney disease (DKD) develops in up to 40% of patients with diabetes and is recognized as a significant microvascular complication of diabetes. Globally, DKD is the primary cause of chronic kidney disease (CKD) and end-stage renal disease (ESRD) (Alicic et al. 2017). DKD is distinguished by microalbuminuria and macroalbuminuria, with morphological changes that include nodular expansion of the mesangium, diffuse thickening of the glomerular/tubular basement membranes, and glomerular hypertrophy (Anders et al. 2018). The pathological features of DKD are characterized by four hierarchies of glomerular lesions, including thickening of the glomerular basement membrane, mild/severe mesangial expansion, nodular sclerosis (Kimmelstiel-Wilson lesions), and glomerulosclerosis (Qi et al. 2017). Hyperglycemia, hypertension, and epigenetics contribute to the promotion of DKD, with its natural course involving glomerular hyperfiltration, progressive albuminuria, a decrease in glomerular filtration rate (GFR), and eventual progression to ESRD (Alicic et al. 2017).

Diabetic kidneys commonly experience hypoxia, and enhancing the activity of hypoxia-inducible factors may present a novel approach to treating DKD by improving kidney oxygen homeostasis (Persson and Palm 2017). Hypoglycemic drugs, such as SGLT2 inhibitors and GLP-1R agonists, along with antihypertensive drugs, contribute to slowing the progression of DKD. However, ineffective treatment or unsatisfactory outcomes are not uncommon (Patel et al. 2020). Addressing this clinical challenge requires the development of innovative strategies to prevent DKD. In the era of big data, Fu et al. (2019) proposed that gaining a new understanding of the changes in kidney structure associated with diabetes, coupled with emerging research technologies, will provide avenues for advancing the clinical treatment of DKD. Timely nephrological evaluations, intensified treatment, and dietary strategies guided by multidisciplinary experts may collectively contribute to the delay in the onset and progression of DKD (Gembillo et al. 2021).

Organic intercommunication between the gut and kidney has been documented to affect human health directly or indirectly (Giordano et al. 2021; Liu et al. 2021). With advances in high-throughput sequencing technologies, the gut microbiota (GM), recognized as a crucial factor in modulating enterocyte metabolism and shaping local/systemic immunity, has gained increasing attention (Krautkramer et al. 2021). Substantial evidence indicates that pathological alterations in the intestinal microbiome during diseases compromise the integrity and function of the intestinal defense barrier, leading to the translocation of viable bacteria from the gut to the extraintestinal kidney. This results in metabolic disorders and the accumulation of uremic toxins (Lv et al. 2022). In contrast, the uremic state of the kidney induces changes in microbial composition, abnormal activation of immune cells, overproduction of antibodies and immune complexes, and infiltration of inflammatory cells, which collectively exacerbates damage to the renal parenchyma (Chen et al. 2019). Modulating abnormal GM is promising as a potential approach to prevent or alleviate kidney diseases related to diabetes and obesity (Zaky et al. 2021).

Clinical and experimental studies on DKD have provided evidence suggesting that novel treatment strategies can be developed once the pathophysiological mechanisms underlying the gut-kidney axis are elucidated (Lehto and Groop 2018; Zhao 2022). Evidence from DKD studies in db/db mice has shed light on the gut-plasma metabolism-kidney axis, and a comprehensive understanding of this axis holds promise for advancing our knowledge of DKD pathogenesis (Wu et al. 2022). Mosterd et al. (2021) summarized evidence on the gut-kidney axis and inflammation driven by gut dysbiosis in CKD and DKD, focusing primarily on short-chain fatty acids (SCFAs) and end-products of protein metabolism. Lu et al. (2018) reported that intestinal dysbiosis activates the renal renin-angiotensin system (RAS), contributing to the development of DKD. Wang et al. (2021) reviewed how GM dysbiosis promotes DKD progression by altering metabolite profiles and activating the immune response. Fernandes et al. (2019) proposed that diabetic GM dysbiosis, characterized by inflammation and immunosenescence, promotes the progression of nephropathy. Lin et al. (2022) demonstrated that the dysbiotic microbiota played a crucial role in the pathogenesis of DKD by generating uremic toxins, emphasizing that modifying the diet and controlling environmental factors are essential strategies to prevent DKD progression. Given the limited attention to the microbial signature and metabolites in animal and human DKD and intervention methods, exploring the potential interaction between the gut and kidney from this perspective is considered a new hypothesis for understanding and managing DKD.

This review presents insights into GM alterations, associated metabolites, and their connections to DKD. We summarize the latest findings concerning the intriguing features of the bidirectional relationship between the gut and kidney, focusing on microbes, metabolite profiling, and signaling molecules. Finally, we discuss and emphasize intervention strategies, including traditional Chinese medicine (TCM) and diet approaches. This study aims to stimulate further research into the intricate pathways associated with the microbiome and its metabolites, offering a promising avenue for treating and managing DKD.

## Materials and methods

### Search strategy

Research questions are as follows: (1) What are the characteristics of gut microbiome in animal and human DKD models? (2) Which metabolites are key indicators of microbial dysregulation in individuals with DKD, and how they change? (3) How does TCM regulate the disordered microbiota in DKD? A comprehensive literature search was conducted in English databases (Web of Science, PubMed, ScienceDirect, Wiley, and Springer Nature), from January 2018 to September 2023, using specific keywords, such as ‘gut microbiota’, ‘diabetic kidney disease’, ‘diabetic nephropathy’, ‘natural product’, ‘traditional Chinese medicine’, ‘Chinese herbal medicine’, and ‘Chinese medicinal herb’. Chinese databases were not searched. The literature retrieval strategy used was: (Topic = ‘gut microbiota’) AND (Topic = ‘diabetic kidney disease’ OR ‘diabetic nephropathy’) AND (Topic = ‘natural product’ OR ‘traditional Chinese medicine’ OR ‘Chinese herbal medicine’ OR ‘Chinese medicinal herb’).

### Inclusion and exclusion criteria

The studies providing the details of microbiome alterations in DKD and of the TCM regulating microbiota were included. Literature containing incomplete references and issue number were excluded.

### Literature screening and data extraction

The irrelevant and duplicate items of the retrieved studies were removed. Two independent investigators (XYH and LZ) first screened studies by titles and abstracts, and further decided the final inclusion by assessing the full texts based on the eligible criteria. The article types of primary research, secondary sources, such as review articles, and randomized controlled trials were included. The following information was extracted: study characteristics, microbial community composition, DKD models, and TCM intervention. Any disagreements were solved by the third investigator (YLZ).

## Results

### Features of the gut microbiome linked to DKD

The primary clinical features of DKD are characterized by varying degrees of albuminuria and damage to the glomerular filtration barrier. However, this paradigm may not fully capture the complexities observed in normoalbuminuric diabetic patients with progressive renal insufficiency and non-proteinuric patients experiencing renal function loss, known as normoalbuminuric DKD and non-proteinuric DKD, respectively. Increasing evidence suggests that the disrupted microbiota and its metabolites, including trimethylamine-*N*-oxide (TMAO), indoxyl sulfate (IS), and *p*-cresol sulfate (p-CS), play crucial roles in DKD (Zhao 2022).

An analysis of fecal samples from 15 nondialysis DKD patients and 15 healthy controls revealed a significant correlation between decreased *Roseburia intestinalis* and increased *Bacteroides stercoris* with clinical indices (HbA1C, total cholesterol) in DKD (Zhang et al. 2022). A meta-analysis involving 15 studies and 1640 participants, including nondialysis DKD patients and healthy controls, showed depletion of *Butyricicoccus*, *Faecalibacterium*, and *Lachnospira* in DKD. In contrast, the genera *Hungatella* and *Escherichia* were significantly enriched, with proportions of statistical differences exceeding 0.8 (Han et al. 2022). A systematic review and meta-analysis of 578 nondialysis DKD patients and 444 healthy controls in 16 studies showed that DKD patients exhibited expansions of the *Escherichia*, *Citrobacter*, and *Klebsiella* genera, along with a depletion of *Roseburia* (Wang et al. 2022c). These findings indicate that patients with DKD exhibit specific alterations in the gut microbiome at various taxonomic levels, emphasizing the importance of understanding taxonomic distributions in different DKD subjects, including the phylum, class, order, family, and genus levels.

Four dominant phyla, Firmicutes, Bacteroidetes, Actinobacteria, and Proteobacteria, make up 99% of bacteria in the healthy human gut (Feng et al. 2019). Firmicutes, as a typical phylum that produces SCFAs, are generally involved in reducing inflammation in the body. Firmicutes mainly include classes Bacilli and Clostridia, orders Lactobacillales and Clostridiales, families Lactobacillaceae and Clostridiaceae, in which genera *Lactobacillus*, *Streptococcus*, *Clostridium* (an opportunistic pathogen), *Ruminococcus*, *Faecalibacterium*, *Oscillospira*, *Roseburia*, *Anaerostipes*, *Dorea*, *Blautia*, and *Lachnospira* are primarily involved (Seong et al. 2018). Lactobacillales are predominated by *Lactobacillus* and *Streptococcus*. Clostridiales account for 95% of Firmicutes. It mainly consists of three common family *Clostridium* clusters: IV (Ruminococcaceae, genera *Ruminococcus*, *Faecalibacterium*, *Oscillospira*, *Anaerotruncus*, *Subdoligranulum*, *Butyricicoccus*), XI (Peptostreptococcaceae, *Clostridium sordellii* and *Clostridioides difficile*, and genus *Anaerococcus*), and XIVa (Lachnospiraceae, genera *Anaerosporobacter*, *Anaerostipes*, *Butyrivibrio*, *Phascolarctobacterium*, *Roseburia*, *Coprococcus*, *Dorea*, and *Blautia*) (Cuív et al. 2015). The genus *Ruminococcus,* which belongs to Ruminococcaceae and Lachnospiraceae, is considered the core bacterium in most human guts, together with *Blautia* (a genus derived from the reassignment of many former *Ruminococcus* spp.), plays a vital role in polysaccharide degradation (La Reau and Suen 2018). Butyrogenic bacteria (*Faecalibacterium*, *Anaerotruncus*, *Subdoligranulum*, *Anaerostipes*, *Butyrivibrio*) within *Clostridium* clusters IV and XIVa are used as probiotics to increase butyrate levels in the colon (Fu et al. 2019). Based on data from experimental murine models of DKD, Li et al. (2020b) presented that the genera *Allobaculum* and *Anaerosporobacter* were more abundant in mice with severe proteinuria. *Blautia* was more abundant in mice with mild proteinuria compared to healthy mice.

The members of Bacteroidetes are known for their ability to degrade a variety of complex carbohydrates. The genera *Bacteroides*, *Prevotella*, *Paraprevotella*, *Porphyromonas*, *Alistipes*, *Odoribacter*, *Parabacteroides*, and *Flavobacterium* are usually predominant (Johnson et al. 2017). *Bacteroides* generally maintain a beneficial relationship with the host. However, they escape the intestinal environment, leading to bacteremia and abscesses (Wexler 2007). In streptozotocin (STZ)-induced diabetic mice, *Bacteroides*, *Alistipes*, and *Odoribacter* x were associated with the progress and therapeutic effect of type 1 diabetes mellitus (T1DM) (Cao et al. 2021). *Prevotella* is classically considered beneficial for health due to its positive role in fermenting a plant-based diet rich in fibers and improving glucose metabolism (Kovatcheva-Datchary et al. 2015). However, some *Prevotella* strains (*Prevotella nigrescens*, *Prevotella intermedia*) are clinically important pathobionts, as they participate in human disease by promoting chronic inflammation (Larsen 2017). The microbial analysis results of 60 nondialysis DKD patients in stages III (urinary albumin to creatinine ratio, UACR 30–300 mg/g), IV (UACR > 300 mg/g) and V (estimated GFR, eGFR < 20 mL/min/1.73 m^2^) showed that the bacterial diversity decreased with DKD progression, and *Bacteroides*, *Alistipes*, *Lachnoclostridium*, *Ruminococcus torques*, *Subdoligranulum* may be detrimental factors of the disease progression (Zhang et al. 2022).

### Relationship of the gut microbiome and its metabolites with DKD

The dysbiosis of the intestinal microbiota is associated with endotoxemia, disruption of the intestinal barrier, abnormal production of metabolites, and inflammatory status (Chi et al. 2021). This dysbiosis has direct effects on renal function. In STZ combined with uninephrectomy-induced DKD rats, positive correlations were observed between Bacteroidetes and Elusimicrobia with UACR and tubular injury index. At the same time, Firmicutes and Actinobacteria were negatively correlated with these indicators (Zhao et al. 2020). In Shenyan Kangfu tablet-treated db/db mice, Chen et al. (2021) found that high abundances of Bacteroidetes and Proteobacteria were positively correlated with HbA1c, urine microalbuminuria, and UACR levels. At the same time, Firmicutes exhibited negative correlations with these three parameters. In resveratrol-treated db/db mice, reversing the altered gut microbiota and inflammatory responses was associated with improved kidney function (Cai et al. 2020). These findings suggest a positive correlation between intestinal microbiota imbalance and DKD.

Microbial dysregulation-associated metabolites have been observed in DKD. Plasma analysis of early-stage DKD patients revealed that producing uremic solutes and oxidative stress markers (glutamine, phenylacetylglutamine, and IS) could indicate declined kidney function (Tan et al. 2021). Barrios et al. (2015) found that IS, phenylacetylglutamine, and p-CS were early markers of kidney function decline and inversely associated with eGFR in fecal samples from 855 individuals with early kidney disease. Gut microbiome-derived phenyl sulfate (PS) led to albuminuria in DKD rats by inducing inflammation and perivascular fibrosis and promoting glomerulosclerosis (Kikuchi et al. 2019). In T2DM-CKD patients, elevated serum LPS had significantly positive correlations with inflammatory marker levels (TNF-α, IL-6) (Salguero et al. 2019). In individuals with DKD, serum and fecal SCFA levels were lower than healthy individuals and negatively correlated with renal function (Zhong et al. 2021). Clinical data from 100 DKD patients showed that acetate, butyrate, and isovalerate were negatively associated with DKD. However, high serum concentrations of propionate and isobutyrate synergistically increased the risk of DKD in T2DM patients (Li et al. 2022). These findings suggest that increased uremic solutes and decreased SCFAs are essential indicators of microbial aberrations in DKD.

**Figure 1.**
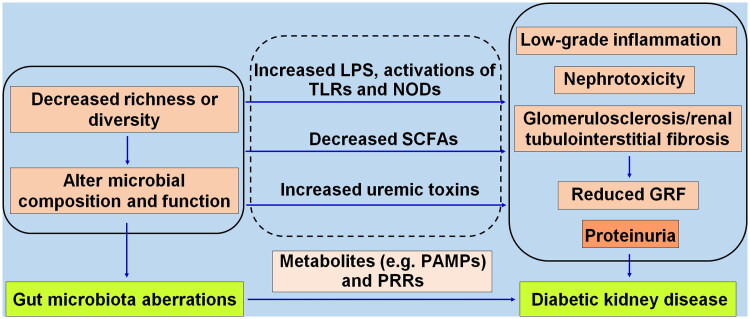
The gut microbiota aberrations promote diabetic kidney disease through metabolic disarrangement involving pathogen-associated molecular patterns (PAMPs) and pathogen recognition receptors (PRRs). The decreased microbial richness or diversity causes alterations in microbial composition and function, translocating opportunistic pathogens (pathobionts) like LPS, and activating toll-like receptors (TLRs), NOD-like receptors (NODs), reducing SCFAs, and increasing uremic toxins, leading to low-grade inflammation, nephrotoxicity, progressive glomerulosclerosis and fibrosis, consequently a reduced glomerular filtration rate (GRF) and proteinuria.

Together, GM aberrations promote DKD by altering its composition and function, accumulating uremic toxins, decreasing the content of beneficial SCFAs, and mediating low-grade inflammation, nephrotoxicity, and glomerulosclerosis/renal tubulointerstitial fibrosis in the kidney. Pathogen recognition receptors (PRRs), as germline-encoded host sensors, receive pathogen-associated molecular patterns in an evolutionarily conserved way and trigger various antimicrobial immune responses (Kumar et al. 2011). Disordered GM promotes DKD through metabolic disarrangement and activation of PRRs. Detailed evidence is shown in Figure 1.

### Gut microbiome-associated metabolites in DKD

GM plays a crucial role in metabolizing complex carbohydrates, proteins, and peptides from food that cannot be digested and absorbed by the small intestine, resulting in the production of a diverse array of end-products, such as SCFAs, amines, indoles, ammonia, thiols, and phenols. The daily diet significantly influences the balance of microorganisms and the production of these metabolites. In both human and animal models of kidney diseases, the accumulation of metabolic endotoxins indicates an increase in proteolytic bacteria (*Enterobacteria* and *Enterococci*) and a reduction in bacteria (*Lactobacillaceae* and *Prevotellaceae*) that possess SCFA-forming enzymes (Lau et al. 2021). Researchers have suggested that SCFAs, bile acids (BAs), and uremic toxins (IS, p-CS, TMAO) play distinct roles in DKD. IS and p-CS are entirely microbial products, while TMAO is derived primarily from the microbiota and dietary components (Mishima et al. 2017). This discussion highlights the characteristics of the main metabolites, aiming to uncover related signaling pathways and gain insight into the intricate communication between the gut and the kidney in DKD.

#### SCFAs

SCFAs, organic fatty acids comprising 1–6 carbon atoms, represent the primary products of bacterial fermentation within the colon. The main SCFAs consist of acetic acid, propionic acid, and butyric acid, with concentration proportions in the colonic lumen of ∼60, 25, and 15%, respectively. Acetate can reach peripheral tissues and undergo metabolic processes in the circulation, while the liver predominantly takes up propionate. Butyrate, a significant energy source for colonocytes and enterocytes, plays a crucial role in regulating the generation, trafficking, and function of adaptive and innate immune cells (Gonçalves et al. 2018). In individuals with advanced DKD, significantly lower levels of valerate and caproate have been shown to predict renal outcomes in patients with biopsy-confirmed DKD (Zhong et al. 2022).

The production of SCFAs constitutes one of the primary mechanisms through which GM modulates the host’s energy metabolism. SCFA content, synthesis, and bioavailability depend highly on dietary components, enzymes, and microbial activity (Koh et al. 2016). Genera, such as *Ruminococcus*, *Faecalibacterium*, *Roseburia*, *Clostridium*, *Coprococcus*, *Blautia*, and *Eubacterium* from the Firmicutes phylum, as well as *Bacteroides*, *Alistipes*, and *Prevotella* within the Bacteroidetes phylum, efficiently release SCFAs by fermenting non-digestible carbohydrates in the upper gastrointestinal tract. Firmicutes primarily produce butyric acids, whereas Bacteroidetes mainly secrete high levels of acetic acid and propionic acid (Fu et al. 2019).

Compelling evidence indicates that SCFAs can directly activate G protein-coupled receptors (GPR41, GPR43, Olfr78, GPR109A), inhibit histone deacetylase, and serve as energy substrates for intestinal cells, positively regulating host physiology. GPR41 and GPR43, highly expressed in adipose tissue and immune cells, respectively, are activated primarily by propionic acid and butyric acid. The affinity values (EC_50_) for propionic acid and butyric acid on GPR41 and GPR43 in humans are 6–127 and 28–371 µM, respectively. Olfr78 is mainly expressed in the renal juxtaglomerular apparatus and is an oxygen sensor for renin secretion and blood pressure regulation. Acetic acid and propionic acid are the primary activators of Olfr78, with EC_50_ values of 2.35 mM and 920 µM, respectively. GPR109A, predominantly expressed in the lumen-facing apical membrane of colonic and intestinal epithelial cells, generally recognizes butyric acid as a ligand, with an EC_50_ value of ∼0.7 mM.

A substantial body of recent research, including experimental animal studies and patient investigations, has shed light on the therapeutic potential of supplemental SCFAs in DKD (Li et al. 2019). Moon et al. (2021) proposed that circulating SCFAs contribute to metabolic processes that mitigate the development of albuminuria in T1DM. Generally, alternative therapy with SCFAs enhances kidney function by activating GPR43 and GPR109A. GPR43 activation promotes the MAPK cascade and inhibits NF-κB activation by coupling with Gi/o and Gq/11 proteins, as well as β-arrestin2, thereby augmenting the immune response in cells. GPR109A plays a role in limiting inflammation by enhancing the anti-inflammatory properties of macrophages and dendritic cells and inducing the differentiation of regulatory T cells (Lymperopoulos et al. 2022).

In the presence of GPR43 or GPR109A, treatment with acetate, butyrate, or propionate protected diabetic mice from nephropathy and inhibited high glucose-induced inflammation in renal tubular cells and podocytes. This protective effect was associated with the suppressed expression of inflammatory cytokines (IL-6, IFN-γ), chemokines (CCL2, CXCL10), and fibrosis-related genes (fibronectin, TGF-β) (Li et al. 2020a). Sodium butyrate (or butyrate) alleviates cellular apoptosis, injury, and inflammation in high glucose-induced cells and db/db mice (Du et al. 2020; Cheng et al. 2022). Exogenous butyrate improved kidney damage in DKD mice and alleviated high glucose-induced injury in glomerular mesangial cells (GMCs) through GPR43-mediated inhibition of oxidative stress and NF-κB signaling (Huang et al. 2020). Supplementary butyrate suppressed the expression of TGF-β and P311 in the kidneys of db/db mice and high glucose-induced SV40-MES-13 cells (Du et al. 2020). In db/db mice, butyrate supplementation in a normal diet protected DKD-induced muscle atrophy by enhancing gut barrier function and activating the GPR41-mediated PI3K/Akt/mTOR pathway (Tang et al. 2022). Cai et al. (2022) found that oral butyrate supplementation improved renal injury in diabetic rats by increasing autophagy-associated activation of the AMPK/mTOR pathway.

Contradictory results regarding the acetate supplementation or the activation of SCFA receptors have emerged. In a DKD model induced by intraperitoneal injection of STZ, acetate exhibited a positive correlation with the expression of the intrarenal angiotensin II protein, and the excessive presence of acetate in plasma-activated intrarenal RAS, which is a known initiator of DKD (Lu et al. 2020). Furthermore, in fecal samples of diabetic rats, excessive acetate disrupted cholesterol homeostasis by activating GPR43, leading to tubulointerstitial injury in DKD (Hu et al. 2020). A previous study also reported that acetate promoted podocyte injury by stimulating GPR43 activation and inhibiting Akt signaling (Lu et al. 2021). In contrast, in GPR109A^-/-^ diabetic mice, GPR109A deletion did not play a critical role in maintaining intestinal homeostasis (Snelson et al. 2020). In summary, these data suggest that the potential beneficial effects of supplementing SCFAs or activating their receptors on DKD remain subject to further investigation.

#### Bile acids

BAs play a crucial role as intermediates in the physiological conversion of lipids, including sterols and phospholipids. Under normal physiological conditions, anaerobic bacteria, such as *Clostridium*, *Bacteroides*, *Enterobacter*, *Escherichia*, and *Bifidobacteria* metabolize primary BAs, such as cholic acid and chenodeoxycholic acid, into secondary BAs, including deoxycholic acid, taurodeoxycholic acid, and taurolithocholic acid, through processes, such as deconjugation, dihydroxylation, and epimerization. In a study in which telmisartan vesicles containing bile salts were administered to STZ-induced DKD rats for 4 weeks, improvements were observed in biological indices (creatinine, urea, total urinary protein) and inflammatory parameters (TNF-α, IL-6) (Ahad et al. 2018).

Identifying specific agonists for BA receptors is crucial for understanding the biological mechanisms of BAs in the kidney. Activation of BA receptors, including the nuclear hormone receptor farnesoid X receptor (FXR) and the membrane-bound G protein-coupled BA receptor 1 (GPBAR1, also known as TGR5), holds promise as a therapeutic approach to prevent DKD (Wang et al. 2018). Naturally occurring primary BAs, such as chenodeoxycholic acid and tauroursodeoxycholic acid (THDCA), followed by secondary BAs, such as cholic acid, deoxycholic acid, and lithocholic acid, are believed to be effective ligands for FXR. Deoxycholic acid and lithocholic acid activate primarily TGR5. THDCA can activate highly expressed FXR in tubular cells and induce the expression of FXR-dependent genes (SOCS3 and DDAH1). THDCA-treated db/db mice exhibited reduced glomerular and tubular injuries (Marquardt et al. 2017).

The FXR agonist GW4064 demonstrated beneficial effects on systemic organs (kidney, pancreas, liver, heart) in db/db mice and improved renal lipid metabolism (Han et al. 2021). Treatment of db/db mice with a selective TGR5 agonist resulted in decreased proteinuria, podocyte injury, mesangial expansion, and CD68 macrophage infiltration into the kidney (Wang et al. 2016). Gentiopicroside, the main active secoiridoid glycoside, could prevent renal inflammation in diabetic mice by regulating the TGR5-β-arrestin2-NF-κB signaling pathway (Xiao et al. 2020). These findings suggest that activating FXR/TGR5 may represent an effective therapeutic option to improve renal injury in diabetic animals. However, the causal relationship between receptor activation and efficacy against DKD needs further elucidation.

#### Amino acid-derived catabolites

Amino acids, the fundamental building blocks of proteins, also function as signaling molecules in regulating metabolism. Modifying the free amino acid profile in plasma, considered a prognostic factor, can potentially mitigate or prevent the occurrence of end-stage DKD (Saleem et al. 2019). In diabetes patients at various stages of CKD, a low level of tryptophan (<44.20 μmol/L) has been associated with a rapid decline in eGFR (Chou et al. 2018). Plasma analysis involving 3089 T2DM patients revealed associations between impaired kidney function, branched-chain and aromatic amino acids, and glycoprotein acetyls (Tofte et al. 2020). Recent experimental studies suggest that the presence of disordered GM-derived tryptophan metabolites serves as an indicator of renal injury (Liu et al. 2021). Modifications in amino acids or their metabolism may play a crucial role in predicting the pathophysiology of DKD.

GM plays a crucial role in the initial steps of amino acid catabolism, primarily through catalytic deamination or decarboxylation reactions that yield carboxylic acids and amines. Commonly catabolized amino acids include sulfur-containing amino acids (methionine and cysteine), basic amino acids (arginine, histidine, and lysine), and aromatic amino acids (tryptophan, tyrosine, and phenylalanine). Generally, methionine and cysteine undergo catabolism into hydrogen sulfide (H_2_S) and methyl mercaptan through degrading enzymes (methionine γ-lyase, cysteine desulfhydrase) present in sulfidogenic bacteria, such as *Streptococcus*, *Clostridium*, *Pseudomonas putida*, *Enterobacter*, and *Klebsiella* (Walker and Schmitt-Kopplin 2021). The expansion of sulfidogenic bacteria, such as *Clostridium*, *Pseudomonas putida*, *Enterobacter*, and *Klebsiella* may contribute to DKD by producing excess sulfides (Xu et al. 2017; Gradisteanu et al. 2019; Rysz et al. 2021). The genera *Clostridium*, *Lactobacillus*, *Streptococcus*, *Bifidobacterium*, and *Escherichia* convert basic amino acids into biogenic amines (Pugin et al. 2017). Tryptophan, tyrosine, and phenylalanine are primarily metabolized by intestinal anaerobes (genera *Clostridium*, *Bacteroides*, *Bifidobacterium*, *Lactobacillus*, *Escherichia*) into indolic and phenolic compounds (Debnath et al. 2021). Tryptophan, being the only amino acid with an indole structure, generates metabolites (indole, indolic acid, skatole, and tryptamine) mainly through indole pyruvate decarboxylase and tryptophanase, enzymes present in the genera *Lactobacillus*, *Clostridium*, and *Bacteroides*, as well as *Clostridium sporogenes* and *Ruminococcus gnavus* species (Gao et al. 2018). The *Enterococcus* and *Clostridium* genera typically degrade tyrosine into p-cresol, tyramine, phenols, and p-coumarate. Limited studies have explored phenylalanine-derived metabolites (phenylethylamine, transcinnamic acid) produced by *Clostridium* and *Peptostreptococcus* (Oliphant and Allen-Vercoe 2019). These findings suggest that the *Clostridium* genus may be crucial in affecting amino acid catabolism.

Indole is generally sulfonated by sulfotransferase 1A1 into IS, while *p*-cresol undergoes sulfation to form aromatic p-CS through aryl sulfotransferases (Gryp et al. 2017). Enzymes responsible for indole and p-cresol formation have been identified in the Clostridiaceae, Enterobacteriaceae, and Verrucomicrobiaceae families (Rysz et al. 2021). Elevated levels of IS and p-CS in patients with T2DM progressing to ESRD were associated with enriched microbes, specifically Clostridiaceae, Enterobacteriaceae, and Verrucomicrobiaceae (Fiaccadori et al. 2020). In DKD mice exhibiting renal tubular injury, a significant increase in plasma IS levels was observed (Ahmed et al. 2022). Meprin-β was found to improve diabetic kidney injury, promoting the production of uremic toxins (IS, N-methyl-pyridone-carboxamide) and altering the metabolite balance in the kidney (Gooding et al. 2019). A preliminary study indicated that the accumulation of IS was associated with increased kidney damage (Ellis et al. 2016; Leong and Sirich 2016). The use of AST-120 (Kremezin^®^) suppressed the production of indole and *p*-cresol, thus delaying the progression of renal dysfunction in DKD (Hwang et al. 2019; Sato et al. 2020).

The formation of *p*-cresol sulfate involves mainly the synthesis of phenol by GM, specifically by bacteria belonging to the Clostridiaceae, Bacteroidaceae, and Bifidobacteriaceae families, followed by further transformation in the liver (Thomas et al. 2021). Kikuchi et al. (2019) proposed that PS could be a predictive marker of the 2-year progression of albuminuria in patients with microalbuminuria. Comprehensive analyses of various experimental models (transgenic rats, diabetic mice) and clinical studies involving diabetes patients indicate that reducing circulating PS levels may mitigate the risk of developing DKD (Fiaccadori et al. 2020). Overexpression of the proximal tubular cell transporter SLCO4C is associated with increased PS excretion, and drugs that upregulate SLCO4C may have therapeutic potential for patients with CKD (Toyohara et al. 2009). In the db/db DKD model, inhibitors of the enzyme tyrosine phenol-lyase reduced plasma PS levels and prevented the progression of renal failure (Kikuchi et al. 2019). In conclusion, an in-depth investigation into identifying amino acids and their catabolites is imperative for diagnosing, preventing, and treating DKD.

#### Trimethylamine N-oxide

TMAO is an oxygenated product derived from trimethylamine (TMA), primarily through the action of hepatic flavin-containing monooxygenases, particularly FMO3. Certain members of GM, including the genera *Sporosarcina*, *Citrobacter*, *Providencia*, *Escherichia-Shigella*, and *Klebsiella pneumoniae* species, play a key role in this process, which depends on dietary nutrients, such as tertiary amines (choline, betaine, l-carnitine) (Janeiro et al. 2018). Human and animal studies suggest that the families Deferribacteraceae (phylum Aquificae), Anaeroplasmataceae (phylum Mollicutes), Prevotellaceae (phylum Bacteroidetes), and Enterobacteriaceae are involved in TMA/TMAO production (Velasquez et al. 2016). In a study by Li et al. (2020b) involving male SPF C57BL/6 mice that received fecal microbiota transplantation (FMT) from DKD mice with severe proteinuria, increased abundance of the genus *Anaerosporobacter* could be related to TMAO production. Individuals with T1DM who had higher plasma TMAO concentrations showed associations with increased mortality, cardiovascular disease events, and unfavorable renal outcomes (Winther et al. 2019).

The plasma level of TMAO is associated with CKD in patients with T2DM (Kalagi et al. 2023). In individuals with T2DM and albuminuria, four metabolites of the TMAO pathway serve as risk markers for the deterioration of renal function (Winther et al. 2021). TMAO promotes DKD by facilitating inflammation, oxidative stress, and fibrosis (Yang et al. 2021). Rats with diet-induced DKD (high-fat diet/low-dose streptozotocin) exhibited higher TMAO levels than normal rats, and TMAO treatment exacerbated kidney dysfunction and renal fibrosis by activating NLRP3 (Fang et al. 2021).

In T2DM-CKD patients, elevated serum TMAO levels showed positive correlations with markers of intestinal permeability (zonulin), endotoxin lipopolysaccharide (LPS), and serum biomarkers of inflammatory and endothelial dysfunction (IL-6, TNF-α, ET-1) (Al-Obaide et al. 2017). Potential approaches to managing TMAO include reducing TMA production by controlling intestinal bacteria or inhibiting the hepatic metabolism of TMA into TMAO. A study demonstrated that lowering microbial TMA production with metformin decreased TMAO availability in db/db mice (Kuka et al. 2020). Another study indicated that targeting a TMA-generating enzyme inhibitor ameliorated renal dysfunction in a mouse model of diet-induced obesity by inhibiting TMA production (Sun et al. 2017). Despite extensive research on the harmful effects of TMAO and its management in DKD, potential mechanisms still warrant further investigation.

### The gut-kidney axis as a therapeutic target for DKD

**Figure 2.**
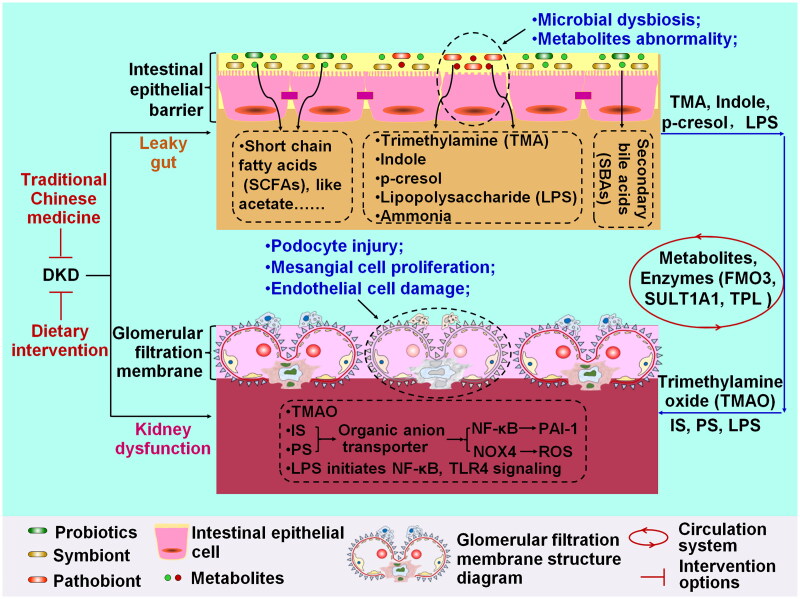
Interpreting the mechanism underlying diabetic kidney disease (DKD) from the interplay between gut and kidney, and the therapeutic target of DKD. GM dysbiosis and the metabolite abnormalities responsible for the leaky gut are important features of intestinal epithelial barrier disruption. Kidney dysfunction characterized by glomerular filtration membrane damage includes podocyte injury, mesangial cell proliferation, and endothelial cell damage. The common metabolites derived from microbiota include SCFAs, trimethylamine (TMA), indole, *p*-cresol, LPS, ammonia, and secondary bile acids (SBAs). TMA, indole, *p*-cresol, and LPS can directly induce gut leakage. In circulation, these compounds are converted into trimethylamine *N*-oxide (TMAO), indoxyl sulfate (is), and phenyl sulfate (PS) by flavin-containing monooxygenase 3 (FMO3), sulfotransferase 1A1 (SULT1A1) and tyrosine phenol-lyase (TPL). When they reach the kidney, is and PS upregulates plasminogen activator inhibitor-1 (PAI-1) expression and increases ROS levels by activating NF-κB and NOX4 signaling. LPS mainly activates NF-κB and TLR4 signaling.

Considering the abnormal communication between the gut and the kidney, disturbances of GM and its metabolites contribute to the exacerbation of kidney dysfunction, mainly manifested by damage to the glomerular filtration membrane (Figure 2). Mitigating deleterious changes in GM composition and metabolite profiles and restoration of impaired glomeruli could be beneficial in improving DKD (Wang et al. 2021; Ni et al. 2022). Potential strategies, including pharmacological therapy and dietary intervention, are summarized in Table 1.

**Table 1.** Summary of intervention approaches targeting interplay of gut and kidney in diabetic kidney disease (DKD).

	Used model	Action mechanism	Treatment	Sample sources	Main metabolites or indicators	References
Pharmacological therapy						
MLB	STZ-induced diabetic DBA/2J mice	Decreasing Clostridiales and Bacteroidales; Improving BA metabolism	Orally administered 50 mg/kg/day MLB for 8 weeks	Feces, urine	Cholic acids and deoxycholic acids	Zhao et al. 2019
TSF	Right uninephrectomy and STZ injection-induced DKD rats	Promoting *Bifidobacteria*; Reducing gut-derived toxins	Oral gavage 1.36 g/kg/day TSF for 12 weeks	Serum, plasma, kidney, feces	IS, LPS	Zhao et al. 2020
QRXZF	HFD and STZ injection-induced DKD mice	Decreasing LPS-producing microbiota; Inhibiting inflammation	Intragastric gavage 15.6 g/kg/day QRXZF for 8 weeks	Serum, urine, kidney and colon tissues, feces	LPS, TLR4 and NF-κB expressions	Gao et al. 2021
SYKFT	db/db mice	Attenuating inflammation; Down-regulating *Bacteroides*	Diets supplemented with 1 and 2 g/kg/day SYKFT for 16 weeks	Blood, urine, kidney, feces	Renal inflammatory molecules	Chen et al. 2021
QDTS	db/db mice	Decreasing Lachnospiraceae_NK4A136_group; Improving BA profiles	Oral gavage 3.37 and 10.29 g/kg/day QDTS for 12 weeks	Serum, urine, kidney and colon tissues, feces	Serum total BA and BA profiles	Wei et al. 2021
SHYS	HFD and STZ-induced DKD mice	Decreasing the Firmicutes to Bacteroidetes ratio; Modulating overall metabolism	Gavage 0.41, 0.81 and 1.62 g/kg/day SHYS for 4 weeks	Serum, kidney tissue, cecum contents	Arginine biosynthesis, TCA cycle, tyrosine metabolism	Su et al. 2021
HKC	Non-obese diabetes mice with DKD	Increasing *Faecalitalea* and *Muribaculum*	Oral gavage with 0.45 g/kg HKC for 4 weeks	Duodenum, ileum and colon	Plasma metabolites	Shi et al. 2022
JSD	STZ-induced DKD mice	Inhibiting kidney injury	Administered 500 mg/kg JSD for 4 weeks	Serum, feces, kidney tissues	Fibrosis regulators (TGF-*β*1 and *α*-SMA)	Meng et al. 2020b
BHID	STZ-induced DKD mice	Acting PI3K/MAPK and MAPK-related protein targets	Administered 100 and 500 mg/kg BHID for 4 weeks	Serum, feces	Fibrosis regulators and inflammatory mediators	Meng et al. 2020a
ZCY	DKD patients based on Chinese DKD guidelines	Improving clinical symptoms	Administered 500 mg/mL ZCY for 8 weeks	Blood, urine, feces, kidney tissue	Serum creatinine, eGFR, clinical symptoms	Liu et al. 2022
RPF	DKD mice	Regulating *Ruminococcaceae_UCG-014, Allobaculum*	Treated with 100 mg/kg/day RPF for 4 weeks	Blood, urine	Ameliorating physiological condition of DKD mice	Zhou et al. 2022
C5aRA	db/db mice; Human glomerular endothelial cells	Activating STAT3 pathway; Disrupting gut-kidney axis	Administered 0.5 mg/kg/day C5aRA for 4 weeks	Serum, urine, kidney, feces	SCFAs, STAT3 pathway	Phull et al. 2021
PFD	db/db mice	Recovering microbial diversity	Administered long-acting PFD for 4 weeks	Blood, urine, cecal	Ketoacidosis biomarkers	Singh et al. 2020
Dietary intervention						
Probiotics based on *L. plantarum*, *Bacillus coagulans*, and *multistrain*	DKD patients aged ≥18 years and disease duration between 2 and 20 years	Improving serum hs-CRP and oxidative stress biomarkers	Supplemented with probiotics from 2.5 to 8 × 10^9^ CFU for 8–12 weeks	Serum	Inflammation and oxidative stress biomarkers	Jb et al. 2021
Probiotic honey containing IBRC-M10791	DKD patients with a proteinuria level > 0.3 g/24 h	Improving serum hs-CRP and plasma MDA levels	Received 25 g/day probiotic honey for 12 weeks	Serum, plasma	Biomarkers of inflammation and oxidative stress	Navid et al. 2019
Probiotic containing ZT-L1, ZT-B1, ZT-Lre and ZT-L3	DKD patients aged 45–85 years with a proteinuria level >0.3 g/24 h	Controlling glycemic level; Reducing cardio-metabolic markers generation	Received 8 × 10^9^ CFU/day probiotic supplements for 12 weeks	Serum, plasma	Metabolic and genetic biomarkers	Mafi et al. 2018
Probiotics *Bifidobacterium bifidum*	DKD patients that have not intake any antidiabetic drugs within 3 months	Reducing HbA1c, serum creatinine and mAlb/Cr	Administrated 3.2 × 10^9^ CFU/day probiotics for 12 weeks	Serum	mAlb/Cr	Jiang et al. 2021
Soy milk containing *L plantarum A7*	Diagnosed with stages 1 and 2 of DKD patients	Improving oxidative stress factors	Administrated 200 mL/day probiotic soy milk	Serum, plasma	Oxidative stress biomarkers	Miraghajani et al. 2017

BUN/CREA, blood urea nitrogen/creatinine ratio; CAs, cholic acids; CFU, colony-forming units; DCAs, deoxycholic acids; eGFR, estimated glomerular filtration rate; HMGB1, High mobility group box protein 1; hs-CRP, high sensitivity-C reactive protein; mAlb/Cr, microalbuminuria/creatinine; MDA, malondialdehyde; TCA cycle, tricarboxylic acid cycle.

#### Pharmacological therapy

Glucose-controlling agents and RAS inhibitors are commonly used in clinical settings to improve kidney function and reduce albuminuria in DKD despite sometimes causing undesirable outcomes. In particular, the SGLT2 inhibitor empagliflozin has been reported to alleviate T2DM-related DKD by reducing LPS-producing bacteria and increasing SCFA-producing bacteria in DKD mice (Deng et al. 2022). Furthermore, emerging studies explore TCM as a potential pharmacological approach for managing DKD (Luo et al. 2021; Cao et al. 2022; Miao et al. 2022; Yu et al. 2022; Wang et al. 2022a, 2022b).

For example, magnesium lithospermate B (MLB), a major component of Danshen water extracts, demonstrated significant decreases in 24 h urinary albumin, bile acids, and the ratio of cholic acid to taurocholic acid when used to treat DKD rats (Zhao et al. 2019). Given that MLB has an exceptionally low bioavailability (0.02%) but a significant oral activity (Lee et al. 2003), the therapeutic impact of MLB on renal injury may be associated with its modulation of the gut microbiome and bile acid metabolic profiles. The Tangshen formula, a traditional Chinese herbal medicine, significantly attenuated renal injury, reconstructed microbial profiles, and decreased metabolic endotoxemia/LPS in DKD rats (Zhao et al. 2020). Similarly, based on the ‘ZhengJia’ theory, the Qing-Re-Xiao-Zheng formula positively affected urinary albumin, colonic mucosa damage, gut dysbiosis, and gut-derived LPS in DKD model mice (Gao et al. 2021).

The Shenyan Kangfu tablet, prescribed to treat CKD, demonstrated alterations in gut microbiota composition by increasing Firmicutes and decreasing Bacteroidetes. Furthermore, it exerted anti-inflammatory activity by downregulating the expression of renal inflammatory markers (NF-κB, TNF-α, and IL-1β) (Chen et al. 2021). The QiDiTangShen granule, formulated based on the theory of ‘filling the renal essence to treat kidney diseases’, reported alterations in gut microbiota composition, with a reduced *Lactobacillus*, *Bacteroides*, and *Lachnospiraceae*_NK4A136_group and an increase in *Alloprevotella*. Additionally, it decreased serum bile acid profiles, and the gut-microbiota-bile acid (GM-BA) axis was presumed to be a crucial target for renoprotection of the QiDiTangShen granule (Wei et al. 2021). The San-Huang-Yi-Shen capsule, used in DKD treatment, ameliorated detrimental effects in DKD rats by improving hyperglycemia, gut microbiota imbalance, renal dysfunction, oxidative stress, and inflammatory response (Su et al. 2021). Similarly, the Huangkui capsule, an *Abelmoschus manihot* (L.) extract, exhibited modulatory effects on the gut microbiota and improved plasma metabolite levels in non-obese diabetic mice with DKD (Shi et al. 2022). These improvements in gut microbiota imbalance and metabolic status, along with the inhibition of inflammatory reactions, are possibly associated with the efficacy of these prescriptions.

Jowiseungki decoction, commonly prescribed for diabetic complications, induced significant changes in the abundance of bacteria, including the Alphaproteobacteria class, the Atopobiaceae family, the genera *Acetatifactor*, *Butyricicoccus*, *Kerstersia*, *Peptococcus*, and *Coriobacteriaceae_UCG-002*, when administered orally to STZ-induced DKD mice (Meng et al. 2020b). The Bekhogainsam decoction, created by Chang ChungChing in the Treatise on Febrile Disease, demonstrated the ability to suppress inflammatory reactions and reshape gut homeostasis in DKD mice. It achieved this by enhancing the growth of probiotic bacteria (*Clostridiales*, *Peptococcus*) and reducing the abundance of *Actinobacteria* (Meng et al. 2020a). Zicuiyin decoction, originating from the Qing dynasty and created by Xichun Zhang, showed the ability to improve kidney function and modulate gut microbiota dysbiosis, particularly in patients with decreased eGFR (Liu et al. 2022). When subjected to solid-state fermentation, the medicinal and edible fungus *Paecilomyces cicadae* significantly reduced urine protein in DKD mice and improved their physiological condition. This effect was attributed to regulating abundances of *Ruminococcaceae_UCG-014*, *Allobaculum*, *Unclassified_f_Lachnospiraceae Alloprevotella*, and *Bacteroides* (Zhou et al. 2022).

Furthermore, a C5a/C5a receptor antagonist alleviated renal macrophage infiltration and proinflammatory factor expression while increasing gut microbiota diversity and SCFA levels in db/db mice (Phull et al. 2021). Long-acting pirfenidone reversed gut microbiota dysbiosis in leptin receptor-deficient db/db mice, showing an increase in *Lactobacillus* and *Bacteroides* and a decrease in *Akkermansi*a. These findings provide insight into the potential beneficial effects of long-acting pirfenidone therapy (Singh et al. 2020). These observations suggest that a comprehensive understanding of the efficacy and mechanisms of traditional medicines will serve as the foundation for basic research on preventing and treating DKD.

#### Dietary intervention

Dietary intervention by supplementing microecological preparations, polysaccharides, and polyphenolic compounds is beneficial for DKD. Introducing sufficient amounts of biotics into dietary supplements is critical to rescuing microbial populations, improving renal health in diabetes, and potentially restoring a proper intestinal environment (Paul et al. 2022). Nagase et al. (2022) proposed that prebiotics and/or probiotics could be individually tailored to prevent and/or treat DKD. Dietary probiotics function by selectively stimulating the growth and/or activity of specific microbial genera/species, with common supplements including *Bifidobacterium* (such as *Bifidobacterium bifidum*, *Bifidobacterium longum* NCC 2705), *Lactobacillus*, *Saccharomyces*, and *Streptococcus*. Numerous studies have shown that probiotic consumption reduces blood urea and *p*-cresol concentrations and systemic inflammation and increases *Bifidobacteria* and *Lactobacillus* counts (Lopes et al. 2018).

In clinical practice, probiotics improve glycemia, renal function, and lipid profiles and reduce serum insulin levels (Moravejolahkami et al. 2021). Probiotic-rich foods, such as honey, milk, and yogurt, have been reported to decrease inflammatory and oxidative stress biomarker levels in the serum and plasma of patients with these conditions (Navid et al. 2019; Jb et al. 2021). In a randomized clinical study, consuming *Bifidobacterium bifidum*, *Bifidobacterium bifidum* strain ZT-B1, *Lactobacillus acidophilus*, and *Lactobacillus acidophilus* ZT-L1 improved glycemic control in patients with DKD (Mafi et al. 2018; Jiang et al. 2021). Furthermore, supplementing soy milk with the *Lactobacillus plantarum* A7 probiotic positively affected albuminuria, eGFR, serum creatinine, IL-18, and sialic acid levels in T2DM patients with nephropathy (Miraghajani et al. 2017). Similar benefits were observed in DKD patients who consumed probiotic-containing soy milk (Miraghajani et al. 2019). A meta-analysis highlighted that the beneficial effects of probiotics depended on intervention duration, probiotic dose, and consumption patterns in patients with DKD (Dai et al. 2022). These findings suggest that probiotic supplements benefit DKD patients by reducing uremic toxins, modifying the gut microbiota, and decreasing inflammatory marker levels. However, these favorable effects exhibit heterogeneity, and their reputation is modest. There is still no consensus on the optimal strain or dose of probiotics for individuals with DKD. Future controlled trials with larger sample sizes and longer follow-up periods are necessary to validate the effectiveness of probiotic supplementation (Vlachou et al. 2020).

## Discussion

Both animal models and humans with DKD indicate that the microbial composition has alterations characterized by a low abundance of Firmicutes and high proportions of Bacteroidetes and Proteobacteria. The proteobacteria-dominated microbiome (family Enterobacteriaceae) is characteristically associated with elevated inflammation. A meta-analysis from hundreds of DKD nondialysis patients and data from the experimental murine models of DKD showed that the beneficial microbes are commonly depleted, specifically Clostridiales order, including families Ruminococcaceae and Lachnospiraceae, genera *Roseburia*, *Faecalibacterium. Blautia*, *Butyricicoccus*, *Coprococcus*, species *Roseburia* spp., *Faecalibacterium prausnitzii*, etc. The genera *Odoribacter, Parabacteroides*, *Alloprevotella*, and *Akkermansia* potentially protect DKD. Some pathogenic bacteria (*Escherichia*, *Allobaculum*, *Hungatella*, *Haemophilus*, *Desulfovibrio*) and opportunistic pathogens (*Clostridium*, *Enterococcus*, *Bacteroides*, *Klebsiella*, *Parasutterella*, *Citrobacter*, *Veillonella*) as detrimental factors promote DKD. Due to the limited reported research, the roles of *Alistipes*, *Rikenella*, *Lachnoclostridium*, and *Subdoligranulum* in DKD must be further confirmed. Overall, GM alterations were found in experimental models of DKD, but more studies should be undertaken to characterize general modifications of the gut microbiome in various DKD models.

Data on the gut microbiome-associated metabolites of DKD patients and animal models demonstrate that the increased uremic solutes and decreased SCFAs are key indicators of microbial dysregulation. Their levels are significantly correlated with inflammatory marker levels (TNF-α, IL-6) and kidney function indicators (e.g., eGFR). These suggest that a comprehensive understanding of the metabolites associated with GM may mitigate kidney damage, and provide valuable information on the roles, spatial concentrations, and potential functions of microbial metabolites. Simultaneously, it also could be a cornerstone for improving systemic inflammatory responses and addressing multiple organ dysfunctions in DKD.

TCM emerges as an ideal regulator of intestinal microecology in rats and mice models of DKD, demonstrating beneficial effects on DKD. Formulations, such as Tangshen formula, Qing-Re-Xiao-Zheng formula, Shenyan Kangfu tablet, and Jowiseungki decoction have a good effect in increasing Firmicutes and decreasing abundances of Bacteroidetes and Proteobacteria. Specifically, probiotic Clostridiales order, families Ruminococcaceae and Lachnospiraceae, genera *Alloprevotella* and *Butyricicoccus* increase, whereas genera *Bacteroides* and *Allobaculum* decrease. The alterations of these microbial compositions protect intestinal barrier function, reduce metabolic endotoxins (e.g., LPS) and some inflammatory markers (e.g., NF-κB, TNF-α, and IL-1β) levels, and balance SCFA and BA metabolism. Despite extensive cellular and animal experiments, clinical trials of TCM for DKD treatment are limited. More research is imperative to comprehensively understand their mechanisms of action, ensure safety, and establish effectiveness, thus promoting TCM as a priority strategy for DKD treatment.

This study has several limitations. First, our data may have omitted publications from other databases, such as International Pharmaceutical Abstracts. Furthermore, our study was limited to publications in the English language. Despite these limitations, the findings of our efforts can offer valuable insight and guidance for future research on the prevention of DKD.

## Conclusions

Profound changes in GM structure, the accumulation of uremic toxins, systemic inflammation, and malnutrition are potential contributors to the abnormal interaction between the gut and the kidney, leading to the onset and progression of DKD. Significant associations between the GM and DKD phenotype, along with microbial markers that classify DKD, offer promising avenues for research. Joint efforts in basic and clinical research to identify the richness/diversity, composition, and metabolic features of DKD could provide opportunities to understand the pathogenesis of the disease. Both pharmacological therapy and dietary intervention have shown beneficial effects in improving DKD. Traditional Chinese herbal medicines, including the Tangshen formula, Shenyan Kangfu tablet, QiDiTangShen granule, and Jowiseungki decoction, have shown protective effects on DKD by targeting the gut-kidney axis related to the microbial disorder, its metabolites, and signaling pathways in DKD. Innovative research and preclinical and clinical trials will provide information on the TCM intervention. Additionally, large-sample clinical investigations covering different stages of DKD that integrate multiomics (such as transcriptomics, proteomics, and metabolomics) and molecular biological methods are essential to develop effective approaches to managing DKD. Deciphering the mechanisms underlying DKD through the interrelationship between the gut and kidney and targeting gut microbiota using TCM represents a promising therapeutic strategy for clinically treating DKD.
